# Identification of race-associated metabolite biomarkers for hepatocellular carcinoma in patients with liver cirrhosis and hepatitis C virus infection

**DOI:** 10.1371/journal.pone.0192748

**Published:** 2018-03-14

**Authors:** Cristina Di Poto, Shisi He, Rency S. Varghese, Yi Zhao, Alessia Ferrarini, Shan Su, Abdullah Karabala, Mesfin Redi, Hassen Mamo, Amol S. Rangnekar, Thomas M. Fishbein, Alexander H. Kroemer, Mahlet G. Tadesse, Rabindra Roy, Zaki A. Sherif, Deepak Kumar, Habtom W. Ressom

**Affiliations:** 1 Department of Oncology, Lombardi Comprehensive Cancer Center, Georgetown University Medical Center, Washington DC, United States of America; 2 Department of Biostatistics, Johns Hopkins University Bloomberg School of Public Health, Baltimore, Maryland, United States of America; 3 MedStar Georgetown University Hospital and Georgetown University Medical Center, Washington, DC, United States of America; 4 Department of Chemistry, Addis Ababa University, Addis Ababa, Ethiopia; 5 Department of Microbial, Cellular and Molecular Biology, Addis Ababa University, Addis Ababa, Ethiopia; 6 Department of Mathematics and Statistics, Georgetown University, Washington DC, United States of America; 7 Department of Biochemistry & Molecular Biology, College of Medicine, Howard University, Washington DC, United States of America; 8 Julius L. Chambers Biomedical/Biotechnology Research Institute, North Carolina Central University, Durham, North Carolina, United States of America; Drexel University College of Medicine, UNITED STATES

## Abstract

Disparities in hepatocellular carcinoma (HCC) incidence and survival have been observed between ethnic groups including African-Americans (AA) and European-Americans (EA). The evaluation of the changes in the levels of metabolites in samples stratified by race could provide a snapshot of ethnically diverse disease related pathways and identify reliable biomarkers. In this study, we considered AA and EA to investigate metabolites that may be associated with HCC in a race-specific manner. The levels of 46 metabolites in plasma samples, collected from patients recruited at MedStar Georgetown University Hospital, were analyzed by Agilent GC-qMS in selected ion monitoring (SIM) mode. A least absolute shrinkage and selection operator (LASSO) regression model was applied to select metabolites with significant changes in HCC vs. cirrhosis in three groups: (1) AA and EA combined; (2) AA separately; and (3) EA separately. In addition, metabolites that distinguish HCC cases from cirrhosis in these three groups were selected by excluding those without HCV infection. The performances of the metabolites selected by LASSO in each group were evaluated through a leave-one-out cross-validation. We identified race-specific metabolites that differentiated HCC cases from cirrhotic controls, yielding better area under the receiver operating characteristics (ROC) curve (AUC) compared to alpha-fetoprotein (AFP), the serological marker widely used for the diagnosis of HCC. This study sheds light on metabolites that could potentially be used as biomarkers for HCC by monitoring their levels in high-risk population of cirrhotic patients in a race-specific manner.

## Introduction

Hepatocellular carcinoma (HCC) is the most common type of liver cancer. An estimated 40,710 new cases of liver cancer (including intrahepatic bile duct cancers) will be diagnosed in the US during 2017, approximately three-fourths of which will be HCC [1]. Most of the HCC patients are diagnosed at late stage when treatment is no more effective, making HCC the most lethal type of liver cancer with an overall 5-year survival rate of approximately 15% [2]. Worldwide, HCC is the fifth most common cancer and the third leading cause of cancer mortality [3].

Persistent infections by HBV or HCV are the main recognized risk factors for HCC [4–8] with the cancer developing faster once the viral-related cirrhosis is established [9, 10]. Epidemiological studies and clinical trials have identified additional demographic, clinical, pharmacological, genetics and life style factors that further affect the likelihood of HCC and can be used in clinical practice to identify at-risk patients through stratified analysis [11]. Longitudinal analysis of cancer follow-up data, stratified by race, has shown higher incidence and mortality rate in African-Americans (AA) affected by HCC [12, 13]. Few reports have addressed racial differences in survival for patients with HCC. In a study where AA and European-Americans (EA) diagnosed with HCC were examined during a 10-year period from 1992 through 2001, it was found that AA were 4% to 20% more likely to die of localized HCC after adjusting for age, sex, and treatment status [14, 15]. Race/ethnicity-specific differences in disease progression and HCC risk may occur even when the underlying liver disease etiology is the same, necessitating a more aggressive disease management and monitoring among certain patient cohorts [16].

Other than liver imaging, current diagnosis of HCC relies on the measurement of the level of the serum biomarker, α-fetoprotein (AFP). However, when considering AFP levels in patients with HCV infection, AFP appears to be insensitive for the diagnosis of HCC in AA. In a case-control study of 163 HCC patients with HCV infection and 149 control patients with HCV-related cirrhosis, the sensitivity of AFP for the diagnosis of HCC in AA with HCV infection was reported to be lower than that of patients of all other ethnic groups combined [17]. Thus, serological biomarkers that take into account differences due to race are highly desired.

Metabolomics has been broadly used for biomarker discovery for many human diseases including cancer [18]. Metabolites, end products of intercellular pathways can potentially serve as indicators of the overall physiological status as well as the response to host and environmental stimuli [19]. Although, it would be difficult to measure concentrations of all metabolites in a biological system by a single analytical method due to their significant chemical diversity and concentration range, the recognition of cancer metabolism (other than somatic mutation) as a hallmark of cancer as first identified by Otto Warburg [20], makes the utility of metabolomics indispensable for the study of cancer biology and hepatocellular carcinogenesis. Considering the disparities that exist for liver diseases and HCC, the evaluation of the changes in the levels of metabolites in samples from a homogeneous racial group could lead to the identification of more reliable race-specific biomarkers than those obtained through whole-population-based approaches.

We previously conducted several metabolomic studies aimed at identifying HCC biomarkers in cirrhotic patients [21–26]. Particularly, in a targeted analysis of metabolites in sera from two study cohorts (Egyptian and US) by using multiple reaction monitoring (MRM), we evaluated HCC biomarkers through stratified analysis by race, gender and alcohol cirrhosis. Two metabolites (3sulfo-glycochenodeoxycholic acid and 3β, 6β-Dihydroxy-5β-cholan-24-oic acid) were selected based on their significance to both cohorts. While both metabolites discriminated HCC cases from cirrhotic controls in males and EA, they were insignificant in females and AA. 3sulfo-glycochenodeoxycholic acid was significant in patients with alcoholic cirrhosis and 3β, 6β-Dihydroxy-5β-cholan-24-oic acid in non-alcoholic cirrhosis, which may also include non-alcoholic steatohepatitis (NASH). These analyses revealed that those clinical covariates are important factors in biomarker discovery.

In this paper, we investigate race-stratified analysis of plasma metabolites measured by gas chromatography coupled with selected ion monitoring mass spectrometry (GC-SIM-MS). The plasma samples were collected from patients recruited at MedStar Georgetown University Hospital (MGUH), Washington, DC. We compared the levels of the metabolites in plasma samples from AA and EA to identify those that distinguish HCC cases from the cirrhotic controls in a race-specific manner. A panel of metabolites was selected using least absolute shrinkage and selection operator (LASSO) logistic regression [27] by considering data from the following groups: (1) AA and EA combined with and without adjustment for race; (2) AA only; and (3) EA only. The analyses were repeated by considering only those with HCV (HCV+). We observed that the combination of LASSO-selected metabolites in each group leads to better prediction in distinguishing HCC cases from cirrhotic controls, compared to AFP. In parallel, a multiple support vector machine recursive feature elimination (MSVM-RFE) [28] model was applied to rank the metabolites in each group, considering only those that are HCV+. While LASSO is used for feature selection by penalizing some features, MSVM-RFE ranks the features allowing better prediction performance than LASSO by controlling the number of selected features [29]. We found that the top metabolites in MSVM-RFE were also selected in LASSO, so the overlapping ones between the two methods were individually compared with AFP.

The discovery of exclusive biomarkers in HCC is still a formidable task mainly due to the heterogeneity of the clinical symptoms of cancer and the various etiologic agents that initiate the pathological liver disorders such as abnormal structural nodules with peripheral fibrosis, cirrhosis, chronic inflammation and fatty liver disease that are precursors to end stage liver disease. To our knowledge, this is the first metabolomic study that seeks to identify race-specific biomarkers by comparing the levels of plasma metabolites in HCC cases against cirrhotic patients by stratifying AA and EA. Replication of these findings may contribute to understanding of the racial disparity in HCC and also in improving diagnosis of HCC through race-specific biomarkers.

## Materials and methods

Adult patients were recruited from the Hepatology Clinic at MedStar Georgetown University Hospital (MGUH). All participants provided informed consent to a protocol approved by the Institutional Review Board (IRB) at Georgetown University. The patients were diagnosed to have liver cirrhosis on the basis of established clinical, laboratory and/or imaging criteria. Cases were diagnosed to have HCC based on well-established diagnostic imaging criteria and/or histology. Clinical stages for HCC cases were determined based on the tumor-node-metastasis (TNM) staging system. Controls were required to be HCC free for at least 6 months from the time of study entry. Race information was collected from patients’ self-report. The characteristics of AA and EA, selected from these patients, are summarized in Table 1, whereas the characteristics of AA and EA that are HCV+ are shown in Table 2. The HCV+ participants were predominantly genotype 1a and 1b with no statistically significant difference between AA and EA.

**Table 1:** Characteristics of AA and EA.

		AA		EA		p-values*		
		HCC (N = 11)	CIRR (N = 8)	HCC (N = 19)	CIRR (N = 31)	AA vs. EA	HCC (AA vs. EA)	CRR (AA vs. EA)
**Age**	**Mean (SD)**	60.6 (4.4)	59.8 (5.2)	57.7 (6.1)	57.8 (6.7)	0.0932	0.0961	0.2280
**Gender**	**Male**	63.6%	87.5%	78.9%	71.0%	1.0	0.4170	0.6530
**HCV Serology**	**HCV Ab+**	100.0%	87.5%	52.6%	41.9%	0.0002	0.0110	0.0440
	**HCV RNA+**	100.0%	87.5%	47.4%	41.9%	0.0001	0.0041	0.0440
**HBV Serology**	**anti HBC+**	63.6%	25.0%	26.3%	29.0%	0.1580	0.7360	1.0
	**HBs Ag+**	9.1%	0.0%	5.3%	3.2%	1.0	1.0	1.0
**MELD**	**Mean (SD)**	9.9 (3.1)	15.6 (5.5)	11.6 (3.6)	18.0 (18.9)	0.1850	0.1156	0.3670
	**MELD <10**	63.6%	25.0%	21.1%	9.7%	0.0083	0.0470	0.2680
**AFP**	**Median (IQR)**	37.4 (73.9)	9.3 (283.4)	14.2 (49.4)	3.4 (5.4)	0.2990	0.1148	0.0010
**HCC Stage**	**Stage I**	27.3%		57.9%			0.142	
	**Stage II**	72.7%		42.1%			0.142	

* Fisher exact test was used for categorical variables. Wilcoxon rank sum test was used for continuous variables that do not have a symmetric distribution. T-test was used for continuous variables with symmetric distribution.

**Table 2:** Characteristics of HCV +, AA and EA.

		AA		EA		p-values*		
		HCC (N = 11)	CIRR (N = 7)	HCC (N = 10)	CIRR (N = 13)	AA vs. EA	HCC (AA vs. EA)	CRR (AA vs. EA)
**Age**	**Mean (SD)**	60.6 (4.4)	59.8 (5.2)	57.7 (6.1)	57.8 (6.7)	0.028	0.057	0.074
**Gender**	**Male**	63.6%	100.0%	90.0%	76.9%	0.713	0.311	0.521
**HBV Serology**	**anti HBC+**	63.6%	28.6%	30.0%	53.8%	0.758	0.198	0.374
	**HBs Ag+**	9.1%	0.0%	0.0%	0.0%	0.439	1.000	1.000
**MELD**	**Mean (SD)**	9.9 (3.1)	15.6 (5.5)	11.6 (3.6)	18.0 (18.9)	0.185	0.116	0.367
	**MELD <10**	63.6%	14.3%	10.0%	15.4%	0.036	0.024	1.0
**AFP**	**Median (IQR)**	37.4 (73.9)	9.3 (283.4)	14.2 (49.4)	3.4 (5.4)	0.281	0.174	0.022
**HCC Stage**	**Stage I**	27.3%		60.0%			0.198	
	**Stage II**	72.7%		40.0%			0.198	

* Fisher exact test was used for categorical variables. Wilcoxon rank sum test was used for continuous variables that do not have a symmetric distribution. T-test was used for continuous variables with symmetric distribution.

The levels of 46 metabolites in plasma samples from AA and EA in Table 1, and additional 15 subjects (Asian, Hispanic/Latino, Other), were analyzed by GC-SIM-MS. Details on sample collection, metabolite extraction, GC-SIM-MS data acquisition, pre-processing, and metabolite ID verification, can be found in Di Poto et al. [26]. In the following sections, we summarize the stratified analysis we performed to identify race-associated biomarkers for HCC.

### Statistical analysis

#### Metabolite selection by LASSO

The metabolite levels corresponding to AA and EA were retrieved from the GC-SIM-MS data previously reported [26]. A LASSO regression model was applied to select a set of metabolites for each of three groups: (1) AA and EA combined (with and without adjustment for race); (2) AA only; and (3) EA only, based on their association with HCC or cirrhotic disease status. These analyses were repeated by considering HCV+ AA and EA. For LASSO models, the tuning parameter *λ* was chosen by cross-validation using the R function cv.glmnet. After estimating the coefficient *β* in (I), metabolites could be selected for each cohort [30].

$$minimize-\sum_{n=1}^{N}Y_{i}\left(\beta_{0}+x_{i}^{T}\beta \right)+\text{log}\left(1+e^{\beta_{0}+x_{i}^{T}\beta }\right)+\lambda {\left\|\beta \right\|}_{1}$$ (I)

*Y*_*i*_ is the status of disease, *x*_*i*_ is the matrix of metabolites for each patient and *N* is the number of patients.

#### Metabolite ranking by MSVM-RFE

We used multiple support vector machine recursive feature elimination (MSVM-RFE) model to rank all metabolites. MSVM-RFE computed feature-ranking score of multiple linear SVMs and reported the average ranking for each metabolite [28]. Area under the curve (AUC) of the receiver operating characteristics (ROC) curve was used to decide the cut-off of top metabolites. The individual metabolite, overlapping in the two methods of LASSO and MSVM-RFE, was compared with AFP in terms of fold change (median values), *p* value (univariate t-test), AUC (95%CI).

#### Performance evaluation of predictors

Logistic regression models were built to evaluate the performance of the predictors in HCV+ patients. Leave-one-out cross validation was used to calculate AUC for the ROC curve in logistic regression using a set of metabolites selected in two different ways: (i) selected metabolites from LASSO; and (ii) top-ranked metabolites in MSVM-RFE. The AUC for the ROC curve was then calculated based on testing data in the cross validation within each model. By R package *pROC* [31], the confidence interval of AUC with Delong`s method, sensitivity and specificity with bootstrap method at 0.5 threshold were performed for the three groups (AA and EA with adjustment for race, AA only and EA only), respectively.

## Results

### Performance of AFP

**Fig 1:**
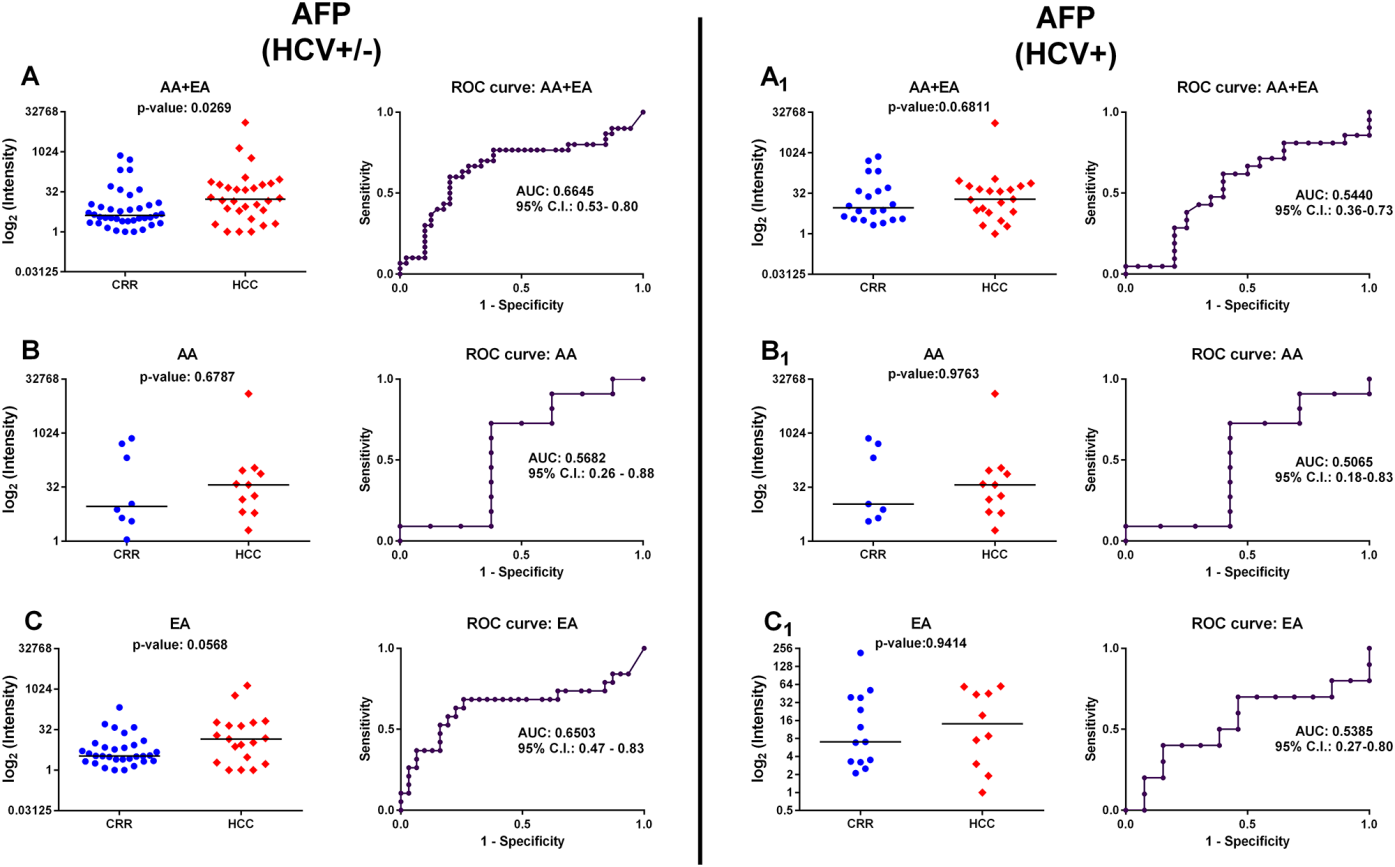
Performance of AFP as a classifier for HCC. The panels show dot plots (circle for liver cirrhotic, diamond for HCC; the horizontal line represents median) and ROC curve (AUC; 95% CI) for AFP for AA and EA combined, AA only, and EA only groups. The left panels (A, B, C) correspond to HCV +/- and the right panels (A_1_, B_1_, C_1_) to HCV+ only.

We evaluated the performance of AFP as a classifier for HCC in AA and EA combined, AA only, and EA only groups, for all subjects (HCV + and HCV-) as well as for HCV+ only subjects. As shown in Fig 1, with the exception of the *p*-value for AA and EA combined (Panel A), AFP was unable to distinguish HCC from cirrhosis in AA (Panel B and B1) or EA (Panel C and C1), emphasizing the need for more potent HCC markers particularly for subjects with HCV+.

### Metabolite selection by LASSO

LASSO regression conducted on AA and EA combined (adjusted or not for race), AA only, and EA only groups, selected a combination of metabolites whose expression levels jointly differentiate HCC cases from cirrhotic controls. The list of metabolites selected in each of the above three groups and considering those with or without HCV infection, is provided in S1 Table. In the comparison between the AA and EA with and without HCV, alpha-D-glucosamine 1-phosphate, palmitic acid, putrescine, tagatose, tyrosine and urea were selected for AA, and of those metabolites only alpha-D-glucosamine 1-phosphate and tyrosine were also selected in the AA and EA combined group when adjusted for race. For EA, glycine, glyceric acid, isoleucine, linoleic acid, oxalic acid, serine, sorbose and threitol were selected; of those, all, except threitol, were selected also in AA and EA combined when adjusted by race. While considering only the HCV+ subjects, palmitic acid, putrescine and tagatose were selected again while ethanolamine and valine were added for AA and of those putrescine and valine were selected in the AA and EA combined group when adjusted for race. For EA, glycine, glyceric acid and threitol were selected again while lactulose, lauric acid, sorbose and tyrosine were added; of those, all metabolites, except lactulose and sorbose, were selected also in the AA and EA combined group when adjusted by race.

### Metabolite ranking by MSVM-RFE

**Fig 2:**
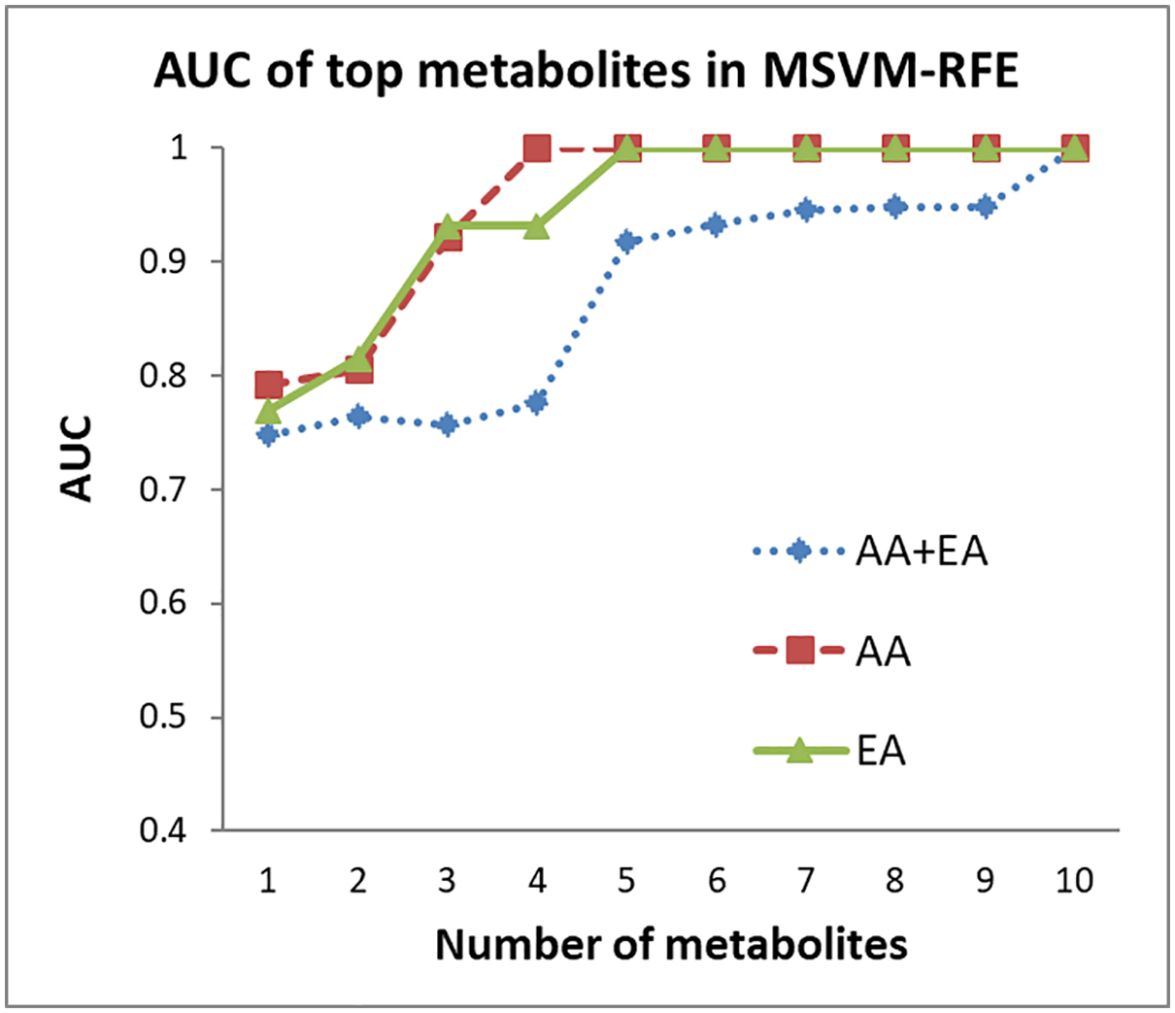
Plot of the AUC values for the ROC curves corresponding to selected top n metabolites (n = 1,…,10), using MSVM-RFE, for AA and EA combined, AA only, and EA only.

MSVM-RFE was used to rank all metabolites. Due to the high percentage of HCV+ in AA, this analysis was conducted for subjects that are HCV+ only. The complete list of the ranked metabolites is provided in S2 Table. The plot of AUC values for the ROC curves using the ranked metabolites, from 1 to 10, from MSVM-RFE are shown in Fig 2. The individual AUC values are provided in the S3 Table. As shown in Fig 2, AUC > 0.9 is achieved with n = 5 metabolites in a panel for all three groups (AA and EA combined, AA only and EA only). The model shows better performance with metabolites selected for AA and EA separately instead of combining them. Specifically, AUC = 0.917 for AA and EA combined, AUC = 1 for AA and AUC = 1 for EA were achieved using MSVM-RFE. The five metabolites selected by MSVM-RFE were also selected by LASSO in all three groups. The metabolites overlapping in the two methods (LASSO and MSVM-RFE) were individually compared against AFP via AUC (Table 3). These metabolites include amino acids and their derivatives (valine, ethanolamine, glutamic acid, phenylalanine, alpha-D-glucosamine 1-phospate, glycine), fatty acids (lauric acid, glyceric acid, linoleic acid) and a vitamin (alpha tocopherol). Among these metabolites, those found to be significant by univariate *t*-test in the comparison between AA and EA within HCC and CIRR groups respectively are indicated. As shown in Table 3, the majority of the metabolites selected in this study showed better AUC in one or all three groups (AA and EA, AA, EA) than AFP. The AUC for valine and alpha tocopherol are the highest in AA only group whereas phenylalanine and glycine in EA. Although AFP tends to have high fold change, it has large variability as shown in Tables 1 and 2. This variability has impacted AFP’s statistical significance and AUC compared to the metabolites listed in Table 3.

**Table 3:** Metabolites selected by both LASSO and MSVM-RFE compared against AFP.

Metabolites	Overlaps: LASSO selected & top 5 MSVM-RFE			FC			*p* value			AUC (95%CI)		
	AA+EA	AA	EA	AA+EA	AA	EA	AA+EA	AA	EA	AA+EA	AA	EA
valine*		✓		↑+1.42	↑+1.69	+1.02	0.054	0.061	0.576	0.67 (0.50,0.83)	**0.79** **(0.57,1)**	0.54 (0.29,0.79)
ethanolamine		✓		↓-1.36	↓-1.57	↓-1.24	0.378	0.655	0.314	0.67 (0.50,0.85)	0.65 (0.34,0.96)	0.63 (0.39,0.88)
glutamic acid**	✓	✓		↓-1.76	↓-1.15	↓-1.73	0.393	0.175	0.549	0.56 (0.38,0.75)	0.64 (0.37,0.90)	0.58 (0.33,0.84)
phenylalanine**			✓	+1.05	↑+1.59	↓-1.64	0.222	0.168	0.010	0.58 (0.40,0.76)	0.69 (0.41,0.97)	**0.77** **(0.57,0.97)**
alpha-D-glucosamine 1-phosphate*			✓	+1.05	↑+7.70	↓-2.18	0.380	0.217	0.725	0.42 (0.24,0.60)	0.69 (0.42,0.96)	0.54 (0.29,0.79)
glycine	✓		✓	↓-1.15	-1.03	↓-1.43	0.054	0.663	0.022	0.65 (0.48,0.82)	0.51 (0.18,0.83)	**0.78** **(0.58,0.98)**
lauric acid**	✓	✓	✓	↓-1.61	+1.01	↓-2.76	0.081	0.504	0.034	0.63 (0.45,0.82)	0.56 (0.24,0.87)	0.72 (0.49,0.94)
glyceric acid	✓			↑+1.29	+1.05	↑1.15	0.753	0.642	0.854	0.53 (0.35,0.72)	0.55 (0.26,0.83)	0.53 (0.28,0.79)
alpha tocopherol	✓	✓	✓	↑+1.46	↑+1.48	↑+1.41	0.015	0.025	0.293	**0.75** **(0.59,0.91)**	**0.82** **(0.60,1)**	0.69 (0.46,0.93)
linoleic acid	✓	✓	✓	↑+1.36	↑+1.37	↑+1.35	0.134	0.184	0.515	0.61 (0.44,0.79)	0.69 (0.43,0.95)	0.55 (0.30,0.80)
AFP	N/A	N/A	N/A	↑+2.10	↑+3.43	↑+2.01	0.680	0.976	0.942	0.54 (0.36,0.73)	0.51 (0.18,0.83)	0.54 (0.27,0.80)

Selected metabolites and AFP along with their fold change (FC), p-value (univariate t-test), and AUC are listed. Fold changes are calculated as HCC vs. cirrhosis. Up-ward arrows indicate metabolites with increased level in HCC vs. cirrhosis (positive FC). Down-ward arrows indicate metabolites with increased level in cirrhosis vs. HCC (negative FC). Metabolites with a small fold change (- 1.10 ≤ FC ≤ + 1.10) are reported without arrow. AUC values ≥ 0.75 are highlighted in bold.

* p<0.05 by univariate *t*-test in the comparison between AA and EA within HCC group.

** p<0.05 by univariate *t*-test in the comparison between AA and EA within CIRR group.

### Performance evaluation of predictors

Leave-one-out cross-validation examined the prediction performance based on these selected metabolites in LASSO and the top-ranked metabolites in MSVM-RFE model. By performing LASSO, 20 metabolites were selected for the AA and EA combined group with adjustment of race factor, 11 for AA and 13 for EA. The top 5 metabolites in all three groups from MSVM-RFE were also selected and evaluated. Table 4 presents the results based on the prediction of the left out sample in the leave-one-out cross-validation where the remaining N-1 samples from the training set were used to estimate the logistic regression coefficients. The performance of the selected metabolites in distinguishing HCC cases from cirrhotic was evaluated using AUC (95% CI), sensitivity and specificity, respectively. As shown in Table 4, the panel of metabolites selected by LASSO, performed well particularly for EA. These metabolites include alpha tocopherol, alpha-D-glucosamine 1-phosphate, glutamic acid, glyceric acid, glycine, lactulose, lauric acid, linoleic acid, oxalic acid, phenylalanine, sorbose, threitol, and tyrosine. The top five metabolites selected by MSVM-RFE, valine glutamic acid, linoleic acid, ethanolamine, and alpha tocopherol, demonstrated better prediction for AA.

**Table 4:** Prediction performance in leave-one-out cross validation based on metabolites selected by LASSO or top five metabolites ranked by MSVM-RFE.

Performance Evaluation	LASSO-selected metabolites			MSVM-RFE-top 5 metabolites		
	AA + EA	AA	EA	AA + EA	AA	EA
**AUC(95% CI)**	0.91(0.83,1)	0.77(0.54,1)	1	0.80(0.65,0.95)	0.87(0.71,1)	0.77(0.59,0.96)
**Sensitivity**	0.86	0.73	1	0.76	0.82	0.7
**Specificity**	0.75	0.86	1	0.7	0.86	0.77

**Fig 3:**
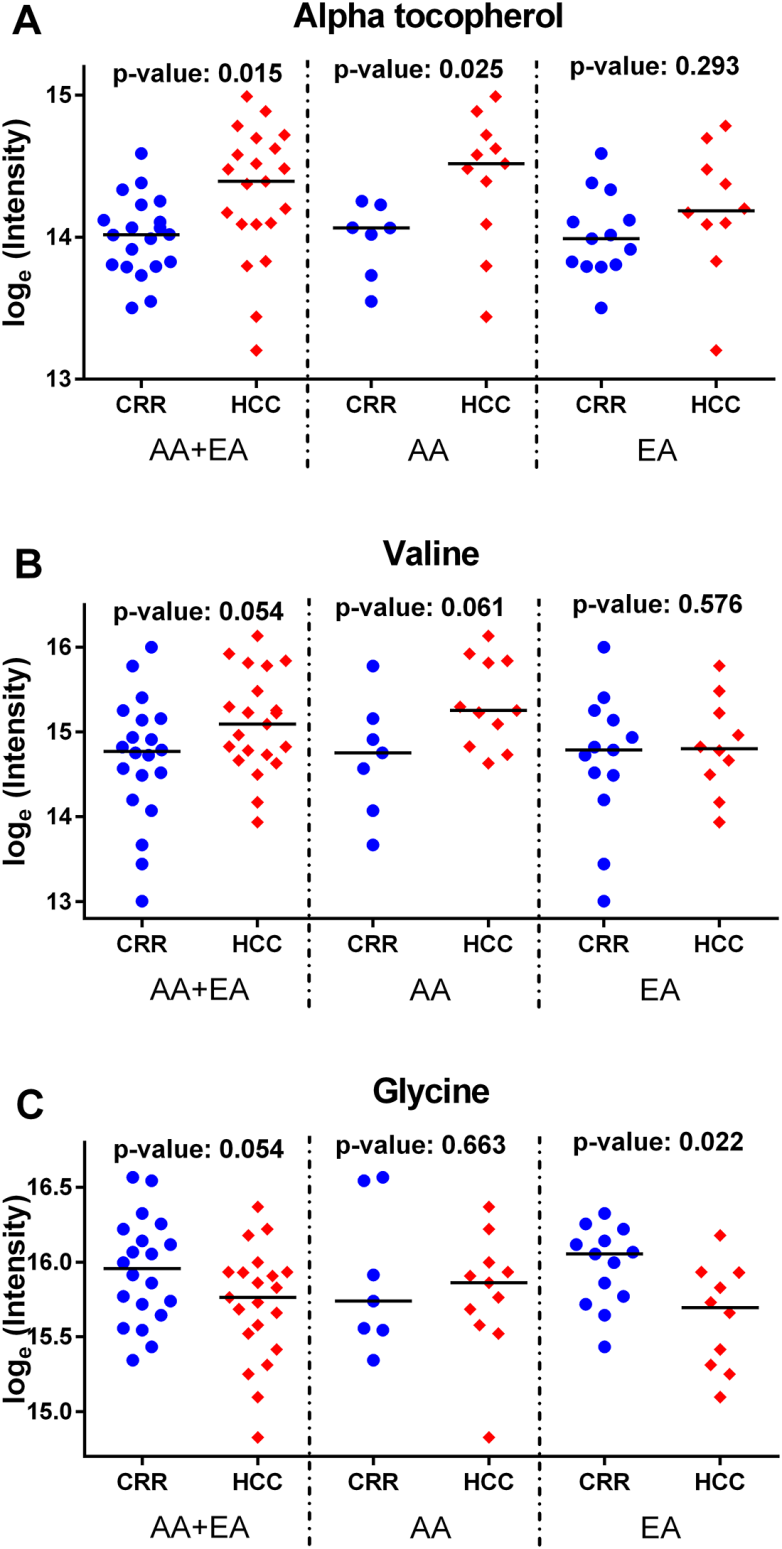
Individual dot plots for alpha tocopherol, valine, and glycine in each group. The individual dot plot, for alpha tocopherol, valine and glycine in AA and EA combined, AA, and EA groups are shown in Fig 3A, 3B, 3C respectively (blue circle dots for liver cirrhotic, red diamond dots for HCC; the horizontal line represents the median level).

**Fig 4:**
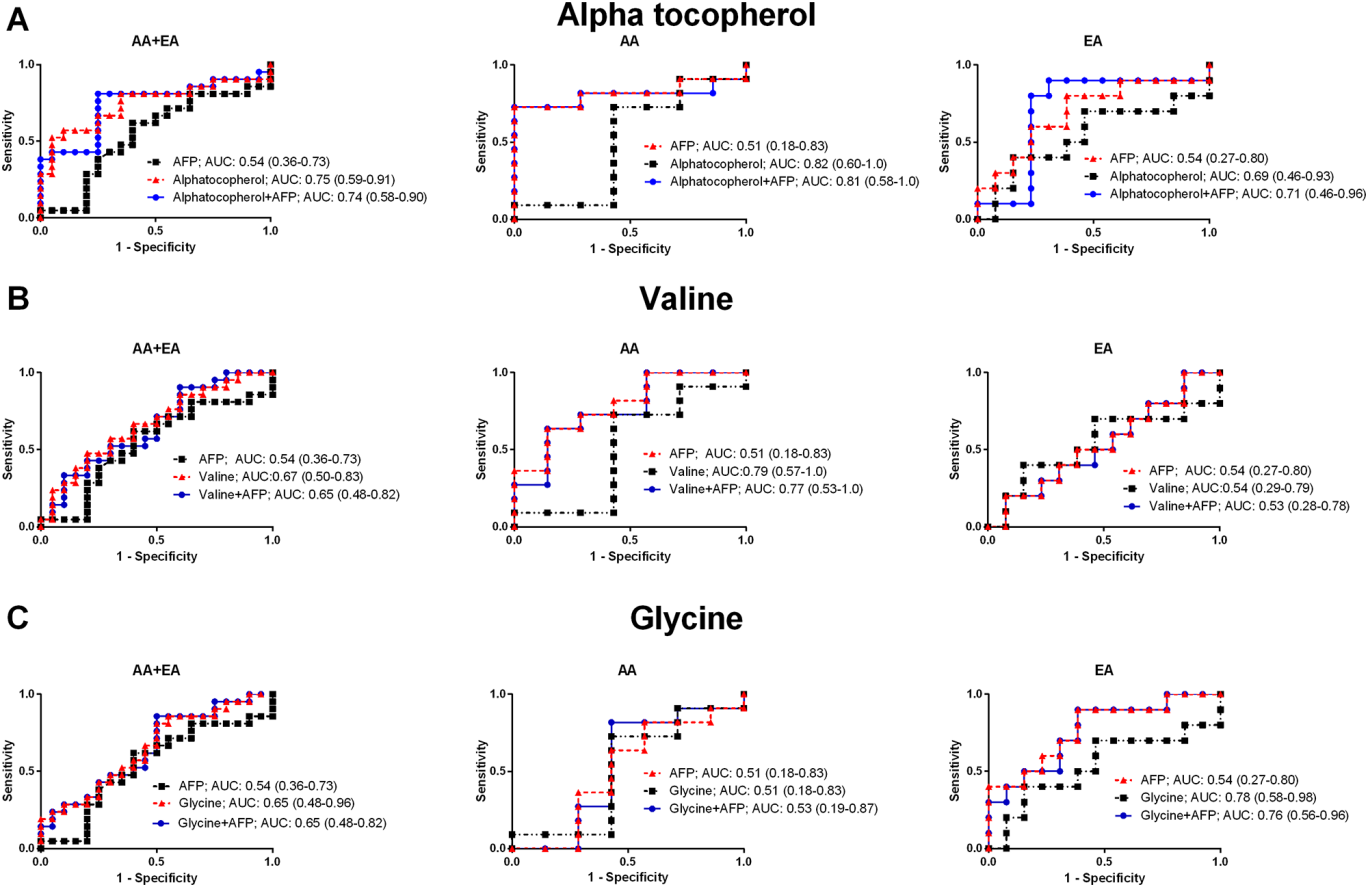
Individual and combined with AFP ROC curves for alpha tocopherol, valine and glycine in each group. ROC curves for alpha tocopherol, valine and glycine in AA and EA combined, AA, and EA groups are shown in Fig 4A, 4B and 4C respectively (black square dot line for AFP, red triangle dot line for alpha tocopherol, valine and glycine and blue circle dot line for the combination of AFP and the corresponding metabolite).

Among the metabolites selected by LASSO regression, those that were top ranked by MSVM-RFE and showed a consistent fold change between HCC cases from the cirrhotic controls across the three groups (AA and EA combined, AA only, and EA only) are: alpha tocopherol, selected in all three groups; whereas valine for AA and glycine for EA. Further biological investigation will focus on them. Fig 3 depicts the individual dot plots for alpha tocopherol (Fig 3A), valine (Fig 3B), and glycine (Fig 3C) in each group (AA and EA combined, AA only, EA only) respectively, showing the changes of the metabolites level from cirrhosis to HCC groups. Furthermore, we looked into the staging for HCC subjects and depicted the correspondent dot plots from cirrhosis, HCC stage I to HCC stage II. These results are available in S1 Fig. In Fig 4 the individual ROC curves for alpha tocopherol (Fig 4A), valine (Fig 4B) and glycine (Fig 4C) and the ones in combination with AFP are shown in each group (AA and EA combined, AA only, EA only), respectively. Candidate metabolites selected in each group (alpha tocopherol for AA and EA combined, valine for AA and glycine for EA) show better performance than AFP only, and the combination with AFP, in distinguishing HCC cases from cirrhotic controls.

To investigate to what degree these metabolites fluctuate in a healthy population, we analyzed sera from the patients presented in Table 1 along with sera from healthy volunteers recruited at MGUH and Howard University Hospital. The levels of valine and glycine were confirmed to have changed significantly in sera from EA vs. AA HCC and cirrhotic patients (p-value = 0.02 for glycine and p-value = 0.009 for valine), whereas alpha-tocopherol had a p-value of 0.08 in EA vs. AA HCC patients. On the other hand, as anticipated, the changes in the levels of these metabolites were statistically insignificant in sera from EA vs. AA healthy volunteers (p-value = 0.65 for alpha-tocopherol, p-value = 0.85 for valine, p-value = 0.35 for glycine). Furthermore, we observed that the variability of the metabolite levels in sera from the healthy volunteers was far less than the variability in sera from cirrhotic and HCC patients, when the healthy and patient groups were frequency-matched by age and gender. This demonstrates that the metabolite levels tend to have less fluctuation in the healthy group compared to the patient groups.

## Discussion and conclusion

The use of metabolomics to identify potential biomarkers of HCC is greatly advantageous to patients and healthcare providers because the dysregulation of metabolites may be an early indication of dysfunctional metabolic pathways that could offer valuable insight into the mechanism of HCC initiation, development or progression. In this study, we investigated plasma metabolites that may be associated with HCC in a race-specific manner by considering AA and EA from a cohort that we previously examined [26]. The levels of selected plasma metabolites were measured by GC-SIM-MS. LASSO regression was conducted to select HCC-associated metabolites in a stratified analysis of AA and EA combined, adjusted or not for race, AA only, and EA only, with or without HCV infection. MSVM-RFE was used to rank the metabolites based on their ability to distinguish HCC cases from cirrhotic controls. The metabolites overlapping between the ones selected by LASSO and the top five ranked by MSVM-RFE were taken into consideration. Several metabolites including alpha tocopherol for AA and EA combined, valine for AA only, and glycine for EA only exhibited better performance than AFP.

AFP has been extensively used as a biomarker for HCC. However, its performance in HCC surveillance has been generally low [32]. In adults, the human AFP gene is silenced by methylation processes and its glycoprotein product reappears only in instances of hepatic damage or tissue regeneration as well as in solid tumors, which makes AFP a non-specific marker for HCC diagnosis [33, 34]. We evaluated the performance of AFP as a classifier (Fig 1). Although in the AA and EA combined group AFP is statistically significant (*p*-value = 0.0269), its performance declines when evaluated in AA only and EA only groups. Gupta *et al*. [35] examined the performance of AFP as a marker for HCC patients with HCV over a period of 30 years. By considering the most commonly reported cut-off for AFP (200 μg/L), they reported AFP’s sensitivity to range from 20% to 45% and specificity from 99% to 100%. The authors stated that AFP appears to have limited utility in identifying HCC in patients with HCV. However, they underlined the limitations of the studies, attesting the need for a prospective study designed to limit bias and define whether a screening strategy can provide clinically important benefits.

We found statistically significant changes in alpha-tocopherol in HCC cases versus cirrhotic controls similar to our previous report [24], which was conducted on an HCV+ Egyptian cohort. Alpha-tocopherol is the most prevalent form of vitamin E that is readily absorbed in mammalian tissues. Many studies including trials have reported the beneficial effects of vitamin E supplementation and higher serum levels of alpha-tocopherol and retinol contributing to increased antioxidant function and reduced risk of liver cancer [36, 37]. This enhanced benefit was also observed in trials involving non-diabetic individuals with NASH (non-alcoholic steatohepatitis) [38] and even became the basis for recent clinical recommendations from the American Association for the Study of Liver Diseases (AASLD) [39]. However, data from recent clinical trials have shown less benefit for alpha-tocopherol in preventing cancer [40–42] or having a prophylactic effect on hepatocarcinogenesis in patients with liver cirrhosis or a chronic HCV infection [43, 44]. The anti-cancer action of this fat-soluble type of vitamin E could be related to its similar uptake and absorption to the dietary cholesterol, preventing the uptake and delivery of the excess of cholesterol to tissues [45]. On the other hand, the abnormal LDL cholesterol metabolism has shown an association with many forms of cancer [46–49]. Therefore, although the variability in the type of cancer, stage of disease and method of treatment in these clinical reports, studying the effect of alpha-tocopherol [40] may be a reason for these controversial results, studies demonstrating the anti-cancer actions of vitamin E should not be disregarded [45].

As candidate biomarkers selected from the analysis based on the larger cohort [26], we reported down- and up-regulation of glycine and valine, respectively, in HCC cases versus cirrhotic controls. In this study, we found that glycine, which is consistently downregulated in HCC cases, is particularly specific to EA whereas valine, consistently upregulated in HCC, is specific to AA. A study conducted on Egyptian subjects, with multivariate statistical analysis of H-NMR spectroscopy data, generated from urine specimen, revealed down-regulation of glycine in HCC cases when compared to cirrhosis subjects [50]. Although these results are not consistent across various studies, as extensively discussed in ref. [50], glycine shows consistency in differentiating patients with tumors. This nonessential amino acid is involved in several synthetic reactions, including protein synthesis, and is also a key component of a central methylation reaction within cells. Its involvement in tumorigenesis could be related to the aberrant DNA methylation mechanism [51]. In contrast, valine, which is an essential amino acid, was shown to occur at higher levels in HCC cases versus cirrhotic controls, and exhibited similar pattern in our previous GC-MS-based study conducted on an HCV + Egyptian cohort [24]. Valine is one of the Branched-chain amino acids (BCAAs). BCAAs levels are carefully regulated by an enzymatic system that quickly responds to conditions of excess or deficiency, playing a crucial role in cancer development [52]. A recent study in hematopoietic stem cell renewal demonstrated that valine plays a crucial role in the creation of blood stem cells and its deficiency or absence in the diet of leukemic mice led to the starvation and death of blood cancer cells [53]. This suggests that the observed increase in the level of valine in the HCC cases may be, in part, due to the steady requirement of this amino acid by the cancer cells for growth and proliferation. Furthermore, not only are metabolites the end products of gene or protein expression, but they are also a manifestation of the relationship that exists between the genome and the internal cellular environment. In that vein, metabolites can be the cause or the result of carcinogenesis in tumor cells such as HCC. Although further studies are warranted for the functional characterization of the metabolic environment and the determination of the relationship between metabolite changes and stage / histologic tumor grade of HCC, identifying critical indicators such as valine may be a significant diagnostic method for the early identification of the disease in clinical practice.

Alpha-tocopherol, glycine and valine were previously reported in HCC related metabolomics studies, conducted by our group and others using additional human specimen, complementary metabolomics platforms and multivariate analysis. In addition, validation of the genetic ancestry of the participants should be performed using a panel of ancestry informative markers. We also acknowledge the possibility of confounding variables other than HCV (such as NASH) for the development of HCC in our study patients.

In summary, we have demonstrated the inability of AFP in discriminating HCC cases from cirrhotic controls, particularly when its performance is evaluated in HCV+ race specific groups. Among the metabolites selected by LASSO, glycine and valine showed better performance than AFP in EA and AA, respectively. Following further validation in a large cohort of patients and healthy controls matched by their demographic characteristics, the metabolites discovered in this study could contribute to better understanding of the development of HCC and allow early detection of HCC in patients with liver cirrhosis and HCV in a race-specific manner.

## Supporting information

S1 Table List of LASSO selected metabolites in each race group and viral infection. (PDF)

S2 Table List of the ranked GC-SIM-MS targeted metabolites by MSVM-RFE in HCV+. (PDF)

S3 Table List of individual AUC values for the ROC curves using the ranked metabolites, from 1 to 10, by MSVM-RFE. (PDF)

S1 Fig Individual dot plots for alpha tocopherol, valine, and glycine in each group. The individual dot plot, for alpha tocopherol, valine and glycine in AA and EA combined, AA, and EA groups are shown in S1A, S1B, S1C Fig respectively (blue circle dots for liver cirrhotic, red diamond dots for HCC; the horizontal line represents the median level). The changes of the metabolites level are shown from cirrhosis, HCC stage I to HCC stage II.
(PDF)
