# Supplementary material for: Identification of race-associated metabolite biomarkers for hepatocellular carcinoma in patients with liver cirrhosis and hepatitis C virus infection
Source: PLoS One. 2018 Mar 14;13(3):e0192748. doi: 10.1371/journal.pone.0192748 (PMC5851549; doi:10.1371/journal.pone.0192748)
Supplement: S1 Table — (PDF) [file pone.0192748.s001.pdf]

S1 Table

		LASSO-selected metabolites		
	**AA and EA**	**AA and EA (adjusted by race)**	**AA**	**EA**
HCV +&-	alpha-D-glucosamine 1-phosphate	alpha-D-glucosamine 1-phosphate	alpha-D-glucosamine 1-phosphate	
	glycine	glycine		glycine
		glyceric acid		glyceric acid
	isoleucine	isoleucine		isoleucine
	pyroglutamic acid/glutamic acid	pyroglutamic acid/glutamic acid	glutamic acid	pyroglutamic acid/glutamic acid
	linoleic acid	linoleic acid		linoleic acid
		oxalic acid		oxalic acid
			palmitic acid	
		phenylalanine	phenylalanine	phenylalanine
			putrescine	
			tagatose	
		tyrosine	tyrosine	
	serine	serine		serine
		sorbose		sorbose
	threitol			threitol
			urea	
	valine	valine 1	valine	valine
		valine 2		
		creatinine		
		arabitol		
		lauric acid		
		alpha tocophereol		
		ethanolamine		
		proline		
		lactulose		
		phosphoric acid		
		trans aconitic acid		
		tyramine		
		glucose		
		glutamic acid		
		arachidic acid		
	**AA and EA**	**AA and EA (adjusted by race)**	**AA**	**EA**
HCV +	alpha tocophereol	alpha tocophereol	alpha tocophereol	alpha tocophereol
	alpha-D-glucosamine 1-phosphate	alpha-D-glucosamine 1-phosphate	alpha-D-glucosamine 1-phosphate	alpha-D-glucosamine 1-phosphate
	arachidic acid	arachidic acid		
	citric acid			
	creatinine			
	cystine			
	ethanolamine		ethanolamine	
	glutamic acid	glutamic acid	glutamic acid	glutamic acid
	glyceric acid	glyceric acid		glyceric acid
	glycine	glycine		glycine
	isoleucine	isoleucine		
	lactic acid			
				lactulose
	lauric acid	lauric acid		lauric acid
	leucine			
	linoleic acid	linoleic acid	linoleic acid	linoleic acid
	N-acetyl-5-hydroxytryptamine	N-acetyl-5-hydroxytryptamine		
	oxalic acid	oxalic acid	oxalic acid	oxalic acid
			palmitic acid	
	phenylalanine	phenylalanine	phenylalanine	phenylalanine
	phosphoric acid	phosphoric acid		
	proline			
		putrescine	putrescine	
			tagatose	
	stearic acid			
				sorbose
	threitol	threitol		threitol
	threonine	threonine		
	tyramine	tyramine		
	tyrosine	tyrosine		tyrosine
	valine	valine	valine	
		pyroglutamic acid/glutamic acid		
