# Supplementary material for: Identification of race-associated metabolite biomarkers for hepatocellular carcinoma in patients with liver cirrhosis and hepatitis C virus infection
Source: PLoS One. 2018 Mar 14;13(3):e0192748. doi: 10.1371/journal.pone.0192748 (PMC5851549; doi:10.1371/journal.pone.0192748)
Supplement: S2 Table — (PDF) [file pone.0192748.s002.pdf]

S2 Table

MSVMR-RFE*

COUNT	AA+EA			AA+EA (Race factor adjusted)			AA			EA	
	**Metabolites**	**Rank**		**Metabolites**	**Rank**		**Metabolites**	**Rank**		**Metabolites**	**Rank**
01	glutamic acid 2	5.286		alpha tocophereol	4.286		valine 1	7.429		phenylalanine 1	5.714
02	alpha tocophereol	5.714		glutamic acid 2	7.429		glutamic acid 2	8.857		linoleic acid	6.143
03	glycine	7.286		linoleic acid	9.286		linoleic acid	9.143		alpha-D-glucosamine 1-phosphate	6.429
04	lauric acid	8.857		glyceric acid	13.000		ethanolamine	10.143		alpha tocophereol	8.000
05	glyceric acid	10.000		lauric acid	13.143		alpha tocophereol	10.429		glycine	8.714
06	linoleic acid	10.571		arachidic acid	13.286		putrescine	12.286		oxalic acid	10.143
07	phosphoric acid	11.714		glycine	14.429		phenylalanine 1	13.000		lauric acid	11.143
08	tyramine	15.571		phosphoric acid	15.429		threonine 2	15.429		tyrosine 2	11.286
09	alpha-D-glucosamine 1-phosphate	15.714		phenylalanine 1	15.571		isoleucine 1	15.714		D threitol	11.429
10	threonine 2	16.143		valine 1	16.714		tyrosine 2	16.000		trans aconitic acid	14.000
11	arachidic acid	16.429		tyramine	17.286		myo inositol	16.429		ethanolamine	14.143
12	proline 2	17.571		threonine 2	17.714		palmitic acid	17.857		glutamic acid 2	14.714
13	isoleucine 2	18.000		pyroglutamic acid/glutamic acid	19.857		sorbose	18.857		urea	19.857
14	valine 1	18.286		tyrosine 2	20.143		citric acid	19.143		sorbose	20.286
15	D threitol	19.143		proline 2	20.429		arachidic acid	19.857		proline 2	20.571
16	phenylalanine 1	21.000		isoleucine 2	21.000		threonine 1	20.143		threonine 1	22.571
17	lactulose 1	21.286		ribitol	21.000		serine 1	21.000		glyceric acid	22.714
18	pyroglutamic acid/glutamic acid	21.571		D threitol	22.429		lactulose 1	23.286		arabitol	24.714
19	ribitol	23.571		serine 1	22.857		tagatose	24.000		arachidic acid	24.857
20	serine 1	23.571		isoleucine 1	23.000		L cystine 3	24.000		isoleucine 2	25.143
21	tyrosine 2	24.429		alpha-D-glucosamine 1-phosphate	23.429		pyroglutamic acid/glutamic acid	24.143		2,3-butanediol	25.429
22	sorbose	25.857		lactic acid	23.429		lactic acid	24.143		tyramine	25.429
23	isoleucine 1	26.000		oxalic acid	24.000		creatinine	24.286		serine 1	25.714
24	glucose	26.000		lactulose 1	24.571		glucose	24.429		isoleucine 1	26.571
25	ribose	26.143		stearic acid	25.143		2,3-butanediol	24.714		myo inositol	27.286
26	oxalic acid	26.286		cholesterol	26.429		oxalic acid	24.857		lactic acid	27.429
27	L cystine 3	26.429		N-acetyl-5-hydroxytryptamine	26.714		D threitol	25.429		ribitol	28.143
28	citric acid	27.857		2,3-butanediol	28.143		phosphoric acid	25.857		creatinine	28.429
29	palmitic acid	28.143		glucose	28.714		tyramine	25.857		citric acid	28.571
30	arabitol	28.286		ribose	29.429		ribitol	27.429		pyroglutamic acid/glutamic acid	28.714
31	valine 2	28.571		D malic acid	29.714		alpha-D-glucosamine 1-phosphate	27.714		tagatose	29.143
32	myo inositol	28.857		trans aconitic acid	29.857		proline 2	28.571		stearic acid	29.286
33	tagatose	29.286		sorbose	30.000		valine 2	28.571		glucose	29.571
34	2,3-butanediol	29.857		creatinine	30.286		N-acetyl-5-hydroxytryptamine	28.571		phosphoric acid	29.857
35	N-acetyl-5-hydroxytryptamine	30.143		valine 2	30.286		isoleucine 2	29.143		putrescine	29.857
36	ethanolamine	30.286		urea	30.571		trans aconitic acid	31.286		threonine 2	30.000
37	creatinine	30.571		myo inositol	30.571		stearic acid	31.857		lactulose 1	30.143
38	cholesterol	30.571		tagatose	31.143		ribose	31.857		leucine 1	31.000
39	D malic acid	32.000		L cystine 3	31.143		glycine	32.000		L cystine 3	31.429
40	urea	32.000		ethanolamine	32.000		cholesterol	32.429		valine 2	32.286
41	stearic acid	32.857		threonine 1	34.571		urea	33.000		D malic acid	32.571
42	putrescine	33.286		leucine 1	34.714		glyceric acid	33.286		cholesterol	32.857
43	leucine 1	34.143		putrescine	34.714		lauric acid	33.571		N-acetyl-5-hydroxytryptamine	33.714
44	trans aconitic acid	34.429		arabitol	35.571		arabitol	34.143		ribose	34.143
45	lactic acid	34.571		citric acid	36.000		leucine 1	35.143		valine 1	35.429
46	threonine 1	36.857		palmitic acid	36.286		D malic acid	35.714		palmitic acid	35.429

*__Metabolites__ sorted from high ranking (lowest value) to low ranking (highest value)
