# Supplementary material for: Identification of race-associated metabolite biomarkers for hepatocellular carcinoma in patients with liver cirrhosis and hepatitis C virus infection
Source: PLoS One. 2018 Mar 14;13(3):e0192748. doi: 10.1371/journal.pone.0192748 (PMC5851549; doi:10.1371/journal.pone.0192748)
Supplement: S3 Table — (PDF) [file pone.0192748.s003.pdf]

S3 Table

AUC			
**# Metabolites**	**MSVM-RFE**		
	**AA+EA**	**AA**	**EA**
**1**	0.748	0.792	0.769
**2**	0.764	0.805	0.815
**3**	0.757	0.922	0.931
**4**	0.776	1	0.931
**5**	0.917	1	1
**6**	0.933	1	1
**7**	0.945	1	1
**8**	0.948	1	1
**9**	0.948	1	1
**10**	1	1	1
