# Supplementary figures and images for: Identification of race-associated metabolite biomarkers for hepatocellular carcinoma in patients with liver cirrhosis and hepatitis C virus infection
Source: PLoS One. 2018 Mar 14;13(3):e0192748. doi: 10.1371/journal.pone.0192748 (PMC5851549; doi:10.1371/journal.pone.0192748)

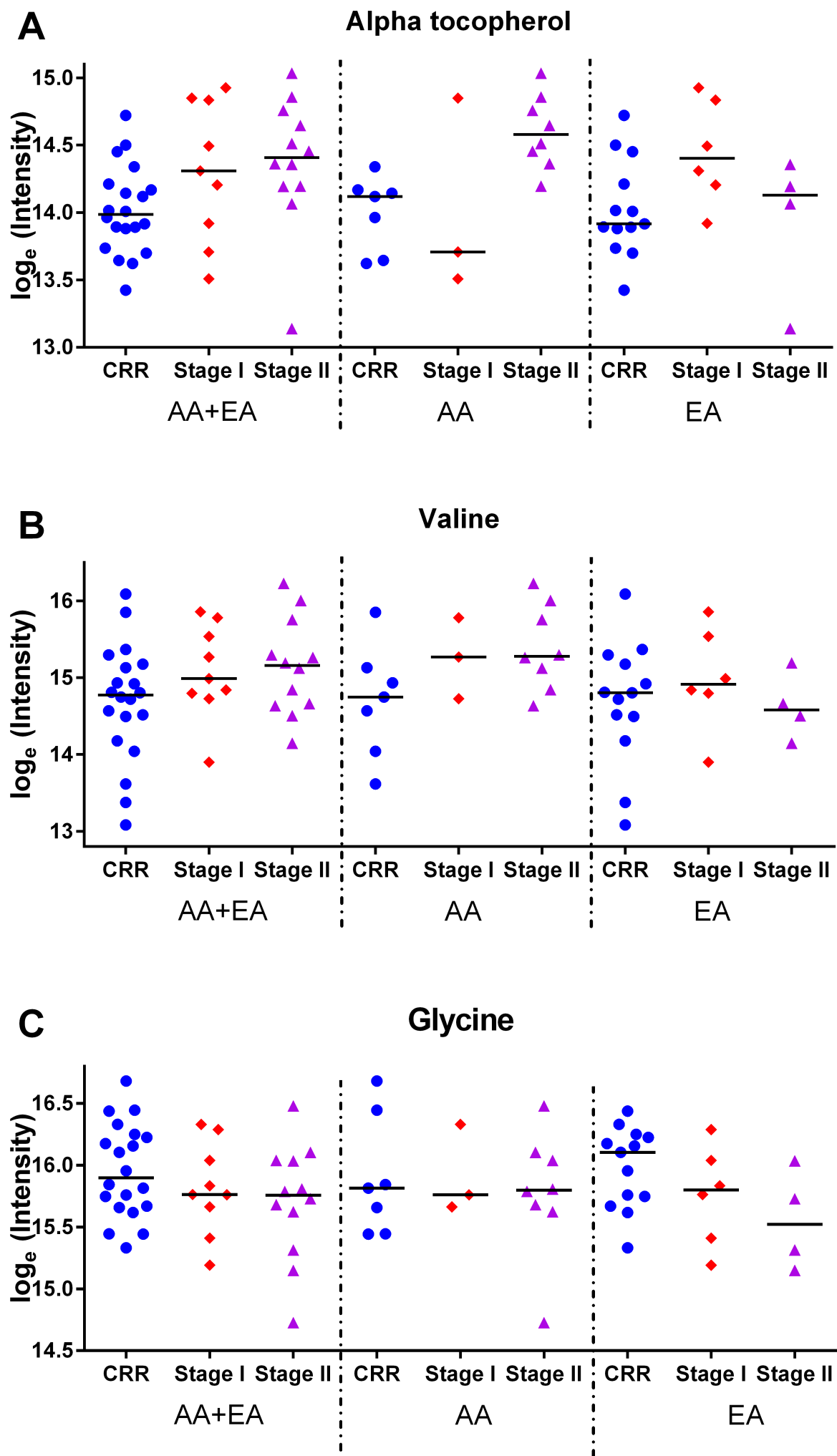

Supplement: S1 Fig — The individual dot plot, for alpha tocopherol, valine and glycine in AA and EA combined, AA, and EA groups are shown in S1A, S1B, S1C Fig respectively (blue circle dots for liver cirrhotic, red diamond dots for HCC; the horizontal line represents the median level). The changes of the metabolites level are shown from cirrhosis, HCC stage I to HCC stage II. (PDF) [file pone.0192748.s004.pdf]
